# Correlation between serum uric acid to high-density lipoprotein cholesterol ratio and atrial fibrillation in patients with NAFLD

**DOI:** 10.1371/journal.pone.0305952

**Published:** 2024-06-24

**Authors:** Gaizhen Liu, Qi Zhang, Meng Zhou, Baojie Li, Jianqi Zhao, Rui Bai, Xiaosu Song, Weiwei Qin, Yonglai Zhang

**Affiliations:** 1 Department of Cardiovascular Medicine, The Second Hospital of Shanxi Medical University, Taiyuan, Shanxi, China; 2 School of Software, North University of China, Taiyuan, Shanxi, China; University of Montenegro-Faculty of Medicine, MONTENEGRO

## Abstract

**Background:**

Non-alcoholic fatty liver disease (NAFLD) is independently associated with atrial fibrillation (AF) risk. The uric acid (UA) to high-density lipoprotein cholesterol (HDL-C) ratio (UHR) has been shown to be closely associated with cardiovascular disease (CVD) and NAFLD. The aim of this study is to clarify whether elevated UHR is associated with the occurrence of AF in patients with NAFLD and to determine whether UHR predicted AF.

**Methods:**

Patients diagnosed with NAFLD in the Department of Cardiovascular Medicine of the Second Hospital of Shanxi Medical University from January 1, 2020, to December 31, 2021, were retrospectively enrolled in this study. The study subjects were categorized into AF group and non-AF group based on the presence or absence of combined AF. Logistic regression was performed to evaluate the correlation between UHR and AF. Sensitivity analysis and subgroup interaction analysis were performed to verify the robustness of the study results. Receiver operating characteristic (ROC) curve analysis was used to determine the optimal cutoff value for UHR to predict the development of AF in patients with NAFLD.

**Results:**

A total of 421 patients with NAFLD were included, including 171 in the AF group and 250 in the non-AF group. In the univariate regression analysis, NAFLD patients with higher UHR were more likely to experience AF, and the risk of AF persisted after confounding factors were adjusted for (OR: 1.010, 95%CI: 1.007–1.013, P<0.001). AF risk increased with increasing UHR quartile (P for trend < 0.001). Despite normal serum UA and HDL-C, UHR was still connected with AF in patients with NAFLD. All subgroup variables did not interact significantly with UHR in the subgroup analysis. The ROC curve analysis showed that the areas under the curve for UA, HDL-C, and UHR were 0.702, 0.606, and 0.720, respectively, suggesting that UHR has a higher predictive value for AF occurrence in NAFLD patients compared to HDL-C or UA alone.

**Conclusion:**

Increased UHR level was independently correlated with a high risk of AF in NAFLD patients.

## Introduction

Atrial fibrillation (AF) is the most common arrhythmia worldwide. With the acceleration of population aging, the prevalence and mortality of AF are increasing year by year [1]. Old age, obesity, dyslipidemia, hypertension (HT), diabetes mellitus (DM), and coronary artery disease (CAD) are recognized risk factors for AF [2,3]. The common pathogenic mechanisms of these risk factors include metabolic disorders, inflammation, oxidative stress (OS), atrial remodeling, and autonomic nervous system imbalance [4,5].

Approximately 25% of the world’s population suffers from Non-alcoholic fatty liver disease (NAFLD), the most common form of chronic liver disease [6]. NAFLD is closely connected with obesity, hyperlipidemia, HT, and hyperglycemia [7–9]. Given the common risk factors between NAFLD and cardiovascular disease (CVD), research suggests that NAFLD is independently linked to AF. In clinical work, we found that many patients with NAFLD complicated with AF, and the serum uric acid (UA) level of patients with AF was generally higher than that of patients without AF, but the high-density lipoprotein cholesterol (HDL-C) level was lower.

The UA to HDL-C ratio (UHR) is a new indicator of metabolic disorders and inflammation, and it is significantly correlated with metabolic syndrome and increased cardiovascular mortality [10,11]. Another study revealed a correlation between elevated UHR levels and the development of NAFLD [12]. Elevated UHR levels increase the risk of NAFLD and CVD. However, it is unclear whether UHR is related to AF risk in NAFLD patients. Hence, the purpose of this cross-sectional study is to determine whether UHR is associated with the occurrence of AF in NAFLD patients.

## Materials and methods

### Study population

Patients diagnosed with NAFLD in the Department of Cardiovascular Medicine of the Second Hospital of Shanxi Medical University from January 1, 2020, to December 31, 2021, were enrolled in the study. We retrospectively included clinical data from these patients. We had access to information that could identify individual participants during data collection by the electronic medical record system. Fatty liver was diagnosed based on liver ultrasound. The occurrence of AF recorded on at least one body surface electrocardiogram or 24-hour holter electrocardiogram during hospitalization or the presence of a clear history of AF was considered AF. The subjects were divided into AF group and non-AF group.

Exclusion criteria: (1) rheumatic, congenital, and valvular heart disease, patients undergoing biological or mechanical valve replacement, hyperthyroidism, or connective tissue disease that may cause AF; (2) viruses, drugs, autoimmune diseases, or other chronic liver diseases; (3) excessive drinking (alcohol equivalent to ethanol amount greater than 70g/ week for women and 140g/ week for men); (4) suffering from severe renal dysfunction; (5) acute infection; (6) lack of clinical data.

NAFLD was diagnosed based on abdominal ultrasound findings by professional operators. And patients lack other causes of hepatic steatosis, such as long-term use of hepatotoxic drugs, excessive alcohol consumption, or a history of viral hepatitis [13].

AF was diagnosed as follows: Standard 12-lead electrocardiogram or ≥30s single-lead electrocardiogram records ECG events with irregular RR intervals and without identifiable P-waves (while not accompanied by atrioventricular block) [1]. AF that terminates automatically or by intervention within 7 days of onset is paroxysmal. AF lasting more than 7 days, including termination by cardioversion (drug or electric cardioversion) after > 7 days, is considered persistent AF.

The local ethics committee of the Second Hospital of Shanxi Medical University approved the study, serial number: (2023) KY NO. (189). Due to the retrospective nature of this study, the need for informed consent was waived by the ethics committee.

### Data collection

Clinical data including age, sex, height, weight, smoking history, HT, DM, and coronary heart disease (CHD) were collected through the electronic medical record system. Collect experimental data, such as white blood cells (WBC), monocytes (MON), lymphocytes (LYM), neutrophils (NEU), hemoglobin (HGB), alanine aminotransferase (ALT), Urea, UA, albumin (ALB), aspartate aminotransferase (AST), triglycerides (TG), total cholesterol (TC), HDL-C, and low-density lipoprotein cholesterol (LDL-C). Blood samples were collected from a fasting venous blood sample taken by a professional the morning after admission.

We calculated the BMI and UHR of all patients. BMI is calculated as weight in kilograms divided by height in meters squared (kg/m2), and UHR is calculated as UA (μmol/L)/HDL-C (mmol/L).

### Statistical analysis

The data was analyzed using R version 4.1.0, GraphPad Prism 9.5.1, and IBM SPSS Statistics 25.0. The Shapiro-Wilk test was used to analyze the normal distribution of continuous variables. Continuous data conforming to normal distribution were represented by mean ± standard deviation, an independent sample t-test was used for inter-group comparison, median (quartile) was represented for non-normal distribution, and a non-parametric test was used for inter-group comparison. Chi-square tests were used to compare groups based on categorical variables. Univariate and multivariate logistic regression were used to evaluate the correlation between UHR and AF. In univariate analysis, variables with p < 0.05 and traditional risk factors for AF were included in multivariate logistic regression analysis. UHR was converted into categorical variables (Q1, Q2, Q3, Q4) according to the UHR quartiles, and the chi-square test was used to compare the multi-group rates. Sensitivity analysis tests were performed to verify whether our results were applicable to people with normal UA and HDL-C. In addition, we performed an interaction analysis that stratified subgroups according to age, sex, and history of HT and CHD to account for the likelihood of different conditions influencing the association between UHR and AF occurrence. Receiver operating characteristic (ROC) curves were used to evaluate the predictive value of UHR for AF occurrence in patients with NAFLD and to determine the optimal cutoff value. P < 0.05 on both sides was considered statistically significant.

## Result

### Comparison of general information

A total of 421 patients with NAFLD were included in our study, including 197 women and 224 men, 171 patients with AF, and 250 patients without AF. The clinical characteristics of the two groups were shown in Table 1. There were no significant differences in BMI, smoking history, WBC, MON, NEU, HGB, ALT, and TG between the two groups (P > 0.05). Age, male, HT, DM, CHD, AST, Urea, UA, and UHR in the AF group were significantly higher than those in the non-AF group, while LYM, ALB, TC, HDL-C, and LDL-C were lower than those in the non-AF group, with statistical significance (P < 0.05) (Table 1).

**Table 1:** Comparison of clinical characteristics between AF group and non-AF group.

	Non-AF (n = 250)	AF (n = 171)	t/χ^2^/Z	P
Age(years)	54.38±11.08	63.91±11.69	-8.475	<0.001
Gender (male)	123(49.2)	101(59.1)	3.969	0.046
BMI(kg/m^2^)	26.40±2.97	26.47±2.74	-0.254	0.800
HT [(n%)]	134(53.6)	117(68.4)	9.265	0.002
DM [(n%)]	38(15.2)	44(25.7)	7.180	0.007
CHD[(n%)]	30(12)	50(29.2)	19.608	<0.001
Smoke [(n%)]	80(32)	62(36.3)	0.823	0.364
WBC(10^9^ /L)	6.35(5.43–7.52)	6.25(5.40–7.39)	-0.502	0.615
LYM(10^9^ /L)	2.00(1.65–2.34)	1.77(1.40–2.32)	-2.835	0.005
MON(10^9^ /L)	0.46(0.37–0.56)	0.47(0.38–0.59)	-0.819	0.413
NEU(10^9^ /L)	3.62(2.91–4.72)	3.75(3.06–4.44)	-0.366	0.714
HGB(g/L)	146.86±14.62	148.24±19.14	-0.798	0.425
ALT(U/L)	21(16.20–31.98)	25.30(16.80–33.40)	-1.548	0.122
AST(U/L)	20.55(17.85–26.13)	22.70(18.60–29.40)	-2.489	0.013
ALB(g/L)	41.42±3.28	40.05±3.80	3.946	<0.001
Urea(mmol/L)	4.90(4.10–5.63)	5.50(4.40–6.80)	-3.893	<0.001
UA(μmol/L)	314.46±73.66	377.94±89.78	-7.651	<0.001
TC(mmol/L)	4.42±0.98	3.90±0.98	5.346	<0.001
TG(mmol/L)	1.60(1.22–2.29)	1.55(1.12–2.06)	-1.559	0.119
HDL-C(mmol/L)	1.10(0.98–1.30)	1.02(0.90–1.20)	-3.712	<0.001
LDL-C(mmol/L)	2.56(2.03–3.01)	2.11(1.66–2.71)	-4.667	<0.001
UHR	276.28(222.22–335.35)	344.44(289.91–453.93)	-7.656	<0.001

Data are displayed as mean ± standard deviation or median and interquartile distance (IQR). P < 0.05 was considered statistically significant. AF, atrial fibrillation; BMI, body mass index; HT, hypertension; DM, diabetes mellitus; CHD, coronary heart disease; WBC, white blood cell count; LYM, lymphocytes; MON, monocyte; NEU, neutrophil; HGB, hemoglobin; ALT, alanine aminotransferase; AST, aspartate aminotransferase; ALB, albumin; UA, uric acid; TC, total cholesterol; TG, triglyceride; HDL-C, high-density lipoprotein cholesterol; LDL-C, low-density lipoprotein cholesterol; UHR, UA to HDL-C ratio.

### Univariate and multivariate regression analysis of AF-related factors in NAFLD

Univariate logistic regression showed that age, gender, DM, HT, CHD, LYM, ALB, Urea, TC, LDL-C, and UHR (OR: 1.009, 95%CI: 1.007–1.012, P < 0.001) were correlated with the occurrence of AF (Table 2). After adjusting for confounding factors, multivariate logistic regression analysis showed that UHR (OR: 1.010, 95%CI: 1.007–1.013, P < 0.001) was still independently related to AF. Age was also a risk factor for AF (OR: 1.077, 95%CI: 1.049–1.106, P < 0.001).

**Table 2:** Univariate and multivariate logistic regression analysis of AF-related factors.

	Univariate		Multivariate	
	OR(95%CI)	P	OR(95%CI)	P
Age(years)	1.078(1.056–1.100)	<0.001	1.077(1.049–1.106)	<0.001
Gender (male)	1.490(1.006–2.207)	0.047	1.196(0.709–2.019)	0.502
BMI(kg/m^2^)	1.009(0.943–1.079)	0.799		
HT [(n%)]	1.876(1.248–2.818)	0.002	1.208(0.731–1.995)	0.461
DM [(n%)]	1.933(1.188–3.144)	0.008	0.904(0.481–1.699)	0.754
CHD[(n%)]	3.030(1.831–5.017)	<0.001	1.457(0.770–2.759)	0.248
Smoke [(n%)]	1.209(0.802–1.821)	0.364		
LYM(10^9^ /L)	0.633(0.459–0.872)	0.005	0.783(0.522–1.174)	0.237
MON(10^9^ /L)	1.539(0.454–5.218)	0.489		
NEU(10^9^ /L)	1.060(0.948–1.186)	0.305		
HGB(g/L)	1.005(0.993–1.017)	0.402		
ALT(U/L)	1.001(0.993–1.010)	0.767		
AST(U/L)	1.012(0.999–1.025)	0.079		
ALB(g/L)	0.893(0.842–0.947)	<0.001	1.010(0.936–1.091)	0.793
Urea(mmol/L)	1.317(1.163–1.492)	<0.001	1.117(0.955–1.306)	0.165
TC(mmol/L)	0.575(0.463–0.713)	<0.001	0.941(0.475–1.864)	0.862
TG(mmol/L)	0.871(0.715–1.061)	0.169		
LDL-C(mmol/L)	0.527(0.394–0.705)	<0.001	0.810(0.324–2.022)	0.651
UHR	1.009(1.007–1.012)	<0.001	1.010(1.007–1.013)	<0.001

### Relationship between UHR quartiles and AF

To further explore the association between different UHR levels and AF in NAFLD patients, we divided participants into four groups based on their UHR values: quartile 1: UHR≤248.00; quartile 2: 248.00 < UHR≤303.88; quartile 3: 303.88 < UHR≤371.88; quartile 4: UHR > 371.88. The prevalence of AF increased with the increase of UHR quartiles. The prevalence of AF in the first group was 20.8%, and in the second, third, and fourth groups was 29.8%, 49.1%, and 62.9%, respectively, with statistical significance (P < 0.001) (Table 3). In the UHR quartiles, we found no association between UHR quartiles and paroxysmal or persistent AF (Table 3).

**Table 3:** The correlation between UHR quartiles and AF in patients with NAFLD.

	Q1(106)	Q2(104)	Q3(106)	Q4(105)	χ^2^	P
AF(%)	22(20.8)	31(29.8)	52(49.1)	66(62.9)	47.038	<0.001
Paroxysmal AF(%)	16(72.7)	16(51.6)	29(55.8)	33(50)	3.616	0.306
Persistent AF(%)	6(27.3)	15(48.4)	23(44.2)	33(50)		

In the unadjusted model, model I, and model II, the risk of AF increased significantly as the UHR quartiles increased (P for trend < 0.001, < 0.001, and < 0.001, respectively) (Table 4). In the unadjusted model, with Q1 as the reference, there was no statistical difference between Q2 and Q1 (P>0.05), and the correlation between the highest quartile of UHR and AF was higher than that between the lowest quartile (OR: 6.462, 95%CI: 3.497–11.940, P<0.001). As Table 4, The OR for Q4 of UHR remained significant in models I and II (OR: 5.859, 95% CI: 2.784–12.330, P<0.001).

**Table 4:** Correlation between UHR quartiles and AF.

	Unadjusted		Model I		Model II	
	OR(95%CI)	P	OR(95%CI)	P	OR(95%CI)	P
UHR	1.009(1.007–1.012)	<0.001	1.010(1.007–1.013)	<0.001	1.010(1.007–1.013)	<0.001
UHR(quartile)						
Quartile 1	Ref		Ref		Ref	
Quartile 2	1.621(0.864–3.044)	0.133	1.432(0.718–2.854)	0.308	1.477(0.732–2.983)	0.276
Quartile 3	3.677(2.009–6.729)	<0.001	3.693(1.877–7.263)	<0.001	3.441(1.715–6.908)	0.001
Quartile 4	6.462(3.497–11.940)	<0.001	6.627(3.253–13.500)	<0.001	5.859(2.784–12.330)	<0.001
P for trend		<0.001		<0.001		<0.001

Model I adjusted for age, gender, DM, HT, BMI, and CHD; Model II adjusted for Model I + LYM, ALB, Urea, TC, and LDL-C.

### Sensitivity analysis

We excluded participants with higher UA levels (> 360 μmol/L in women and > 420 μmol/L in men) and lower HDL-C levels (<1.03 mmol/L) for sensitivity analysis. 179 patients with NAFLD had normal UA and HDL-C, and 52 had comorbid AF. In these patients, multivariate regression analysis showed that higher UHR values were independently associated with the risk of AF (OR: 1.009, 95%CI: 1.002–1.015, P = 0.010) (Table 5). This suggests that despite normal serum UA and HDL-C, UHR was still connected with AF.

**Table 5:** Univariate and multivariate logistic analyses associated with the risk of AF in the normal UA and HDL-C ranges.

	Univariate		Multivariate	
	OR(95%CI)	P	OR(95%CI)	P
Age(years)	1.070(1.033–1.109)	<0.001	1.069(1.031–1.108)	<0.001
Gender (male)	1.819(0.948–3.489)	0.072		
BMI(kg/m^2^)	1.020(0.909–1.145)	0.735		
HT [(n%)]	1.821(0.938–3.534)	0.076		
DM [(n%)]	2.353(0.977–5.665)	0.056		
CHD[(n%)]	2.421(1.034–5.672)	0.042	1.511(0.583–3.916)	0.395
Smoke [(n%)]	1.032(0.489–2.179)	0.934		
LYM(10^9^ /L)	0.641(0.375–1.098)	0.106		
MON(10^9^ /L)	0.332(0.035–3.126)	0.335		
NEU(10^9^ /L)	0.934(0.722–1.208)	0.602		
ALT(U/L)	1.016(0.996–1.036)	0.120		
AST(U/L)	1.021(0.992–1.051)	0.160		
ALB(g/L)	0.982(0.893–1.079)	0.701		
UREA(mmol/L)	1.155(0.939–1.422)	0.173		
TC(mmol/L)	0.661(0.461–0.948)	0.024	0.861(0.558–1.329)	0.499
TG(mmol/L)	0.555(0.313–0.984)	0.044	0.664(0.345–1.275)	0.218
LDL-C(mmol/L)	0.729(0.463–1.148)	0.172		
UHR	1.006(1.001–1.012)	0.027	1.009(1.002–1.015)	0.010

**Fig 1:**
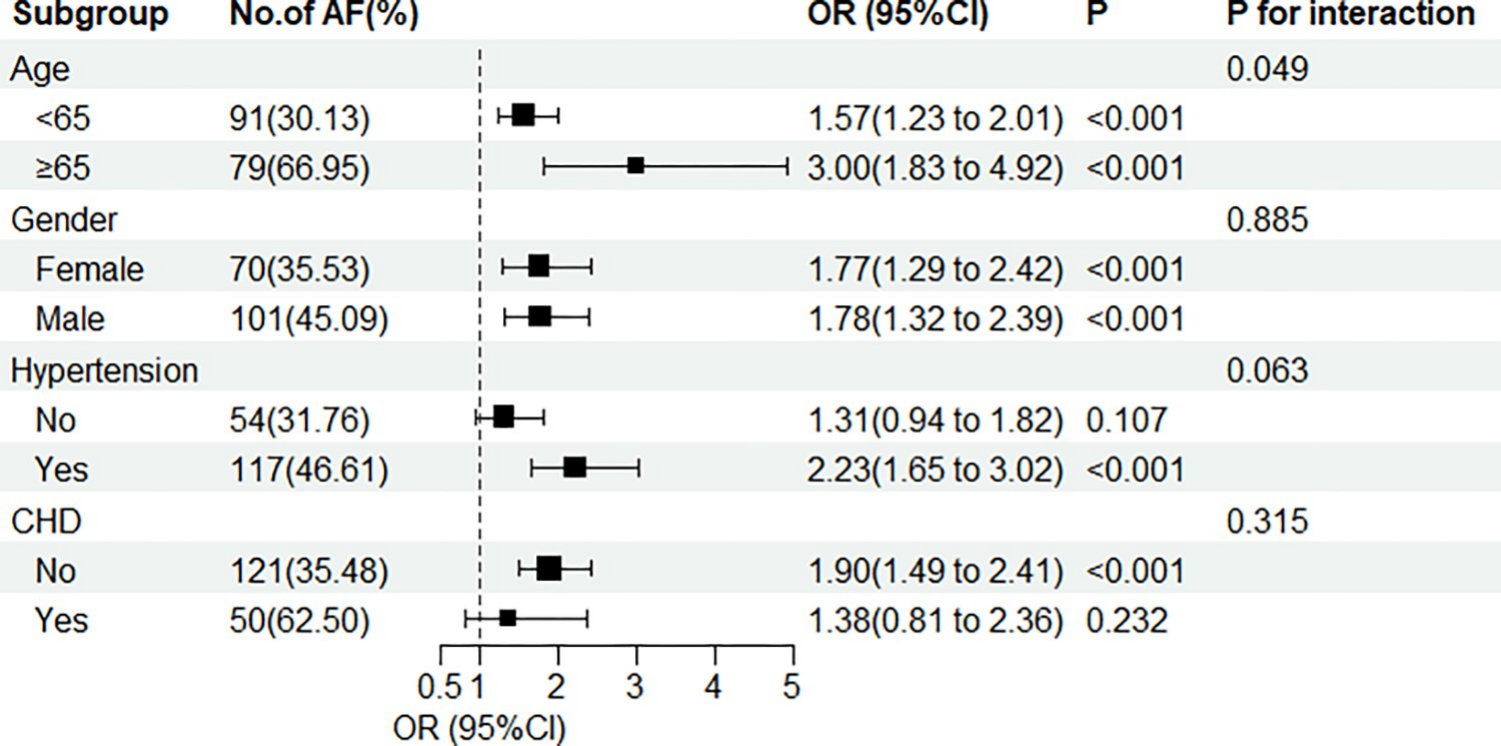
Subgroup analyses. A comparison of the adjusted OR of AF for the subgroups is presented by forest plot. Adjusted for age, sex, HT, DM, and CHD for each subgroup (excluding its own group).

To assess whether subgroups influenced the association between UHR and AF, we performed subgroup stratification analyses based on gender (male or female), age (<65 or ≥65 years), HT (with or without), and DM (with or without) (Fig 1). All subgroup variables did not interact significantly with UHR in subgroup analysis. This suggests that regardless of the above factors, elevated UHR levels increase the risk of developing AF.

### ROC curve analysis

**Fig 2:**
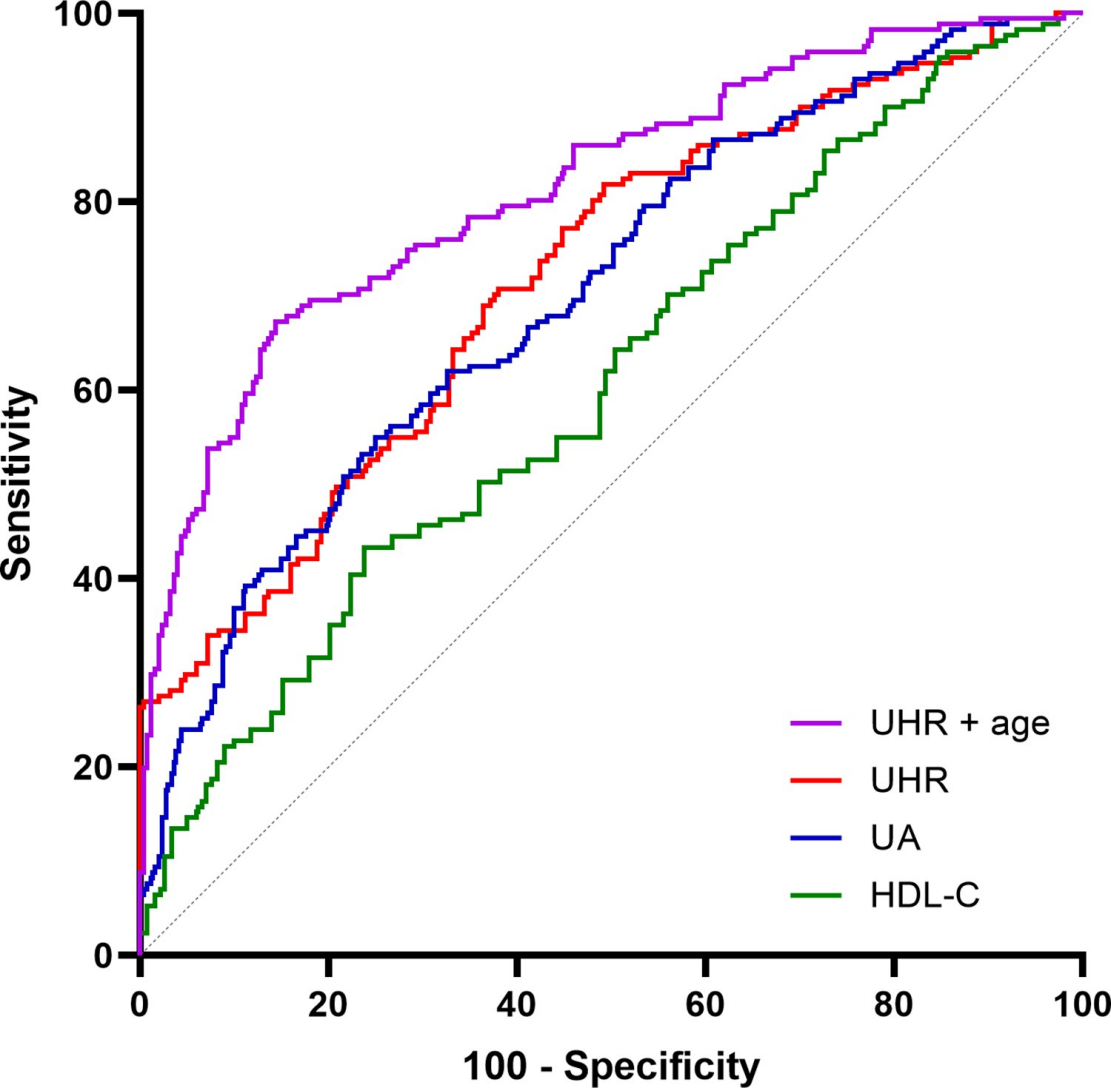
ROC curves.

ROC curves were plotted with UHR, UA, and HDL-C levels respectively to evaluate the predictive ability of AF occurrence in NAFLD patients (Table 6, Fig 2). UA or HDL-C alone has a lower area under the curve (AUC) than UHR, suggesting that UHR has a higher predictive value for AF occurrence in NAFLD patients compared to HDL-C or UA alone. The sensitivity and specificity of UHR levels greater than 300.455 for predicting AF were 70.8% and 62.0% (AUC: 0.720, 95%CI: 0.670–0.769, P<0.001). In addition, the combined age of UHR (0.814 vs. 0.720) further improved the ability to predict AF.

**Table 6:** ROC curve analysis.

	AUC	95%CI	P	Sensibility	Specificity	Cut-off
UHR	0.720	0.670–0.769	<0.001	0.708	0.620	300.455
UA	0.702	0.652–0.752	<0.001	0.550	0.748	356.500
HDL	0.606	0.552–0.661	<0.001	0.433	0.752	0.975
UHR with age	0.814	0.772–0.857	<0.001	0.673	0.856	-

## Discussion

As far as we know, this is the first study to examine the association between UHR and AF in patients with NAFLD. Our study found that UHR values were significantly higher in the NAFLD combined with AF group than in the non-AF group, and that higher UHR levels were associated with a higher risk of AF in the NAFLD population. After adjusting for age, sex, HT, CHD, and DM, this relationship remained. Sensitivity analyses showed that despite normal serum UA and HDL-C, UHR was still correlated with AF in patients with NAFLD. In addition, the interaction between UHR and gender, HT, and DM subgroup variables was not significant in subgroup analysis, but the P for interaction between UHR and age subgroup was 0.049, subgroups had a smaller sample size, which may have contributed to the results, and the chance of a positive result cannot be excluded. Future studies with large sample sizes are still needed to confirm this association. In addition, the ROC curves demonstrated that UHR has a significantly higher predictive value than UA or HDL-C alone for AF, and UHR combined with age could further improve the predictive ability for AF. Our study demonstrates that UHR can be used as an indicator of AF occurrence in patients with NAFLD and becomes a convenient and useful tool for predicting AF.

The UHR is considered a new metabolic and inflammatory marker. The average UHR level is higher in patients with metabolic syndrome [14]. As well as determining individuals at high risk of NAFLD, UHR has been shown to be significantly more predictive than UA and HDL-C alone in non-obese Chinese with normal lipid levels [15]. A recent study showed that UHR was a new marker for predicting coronary artery stenosis [16]. Peritoneal dialysis patients with increased UHR levels had a significantly increased risk of cardiovascular mortality, according to another retrospective cohort study [11]. UHR is associated with other conditions that contribute to AF and coronary vascular diseases. For example, elevated UHR has been reported to be independently associated with poorly controlled HT, with a 7.3-fold increase in the risk of poor blood pressure control for each unit of elevated UHR [17]. Another study reported that elevated UHR was also a biomarker of diabetic kidney injury, with diabetic patients with kidney injury having higher UHR levels compared to diabetic patients without kidney injury [18]. UHR values reflect insulin resistance (IR), and higher UHR values are positively correlated with chronic inflammation and ischemic heart disease events [19]. In addition, UHR has also been shown to be a useful and reliable marker for type 2 diabetes control [20], chronic kidney disease [21], and hashimoto thyroiditis [22]. In summary, these studies suggest that UHR is a valuable predictive tool for metabolic disorders, fatty liver, and CVD. However, the relationship between AF, as a disease closely associated with metabolic disorders, and UHR has not been well studied, and it is unclear whether UHR can predict AF risk in NAFLD patients. The results of this study indicated that UHR was significantly associated with the occurrence of AF in patients with NAFLD.

Under normal concentration, UA has an antioxidant effect, and with the increase of UA concentration, it plays a role in promoting oxidation and becomes a sign of metabolic disorders [23]. A Mendelian randomization study conducted by Li et al [24] showed that serum UA levels were elevated in NAFLD patients. A cohort study of 17,858 participants suggested that there was a significant association between elevated UA levels and CVD death among NAFLD patients [25]. Elevated UA significantly increased liver fibrosis in patients with fatty liver [26]. NAFLD patients have a higher level of UA than people without NAFLD, according to these studies, and UA can predict the degree of fibrosis progression and cardiovascular complications in NAFLD. AF has always been the focus of attention because of its high prevalence and serious complications. There is a strong correlation between high UA levels and AF, even after other confounding factors have been adjusted [27,28]. A nationwide cohort study showed that after adjusting for multiple confounding factors, NAFLD was still positively correlated with AF risk [29]. In conclusion, UA, NAFLD, and AF are closely related, but their potentially related mechanisms have not been fully elucidated. They mainly include metabolic disorders, OS, endoplasmic reticulum stress (ERS), and inflammation.

IR is one of the main mechanisms of metabolic disorders in metabolic syndrome. IR is involved in the development of AF, and the TyG index is a simple and low-cost marker for assessing IR. A systematic review and meta-analysis of 50,921 individuals in nine studies explored the association between TyG and the occurrence of AF [30]. The results found that the prevalence of AF is higher in the group with high TyG levels compared to the low TyG group, and the TyG index is higher in patients with AF compared to the normal population. The TyG index can predict the risk of AF in patients with NAFLD [31]. Our study found that UHR is also a risk factor for the development of AF in patients with NAFLD. The accumulation of lipid intermediates such as diglycerides and ceramides in the fatty liver leads to inhibition of insulin signaling cascade, inducing IR and lipid deposition. Compensatory hyperinsulinemia induced by IR affects the renal transporter of urate, reduces renal clearance of UA, and leads to reduced excretion of UA and increased accumulation of UA [32,33]. Elevated UA levels can induce an increase in monocyte chemotactic protein-1 (MCP-1) production at mRNA expression and protein release levels. MCP-1 is a kind of adipokine, and the increased expression of MCP-1 stimulates the infiltration of macrophages in adipose tissue and hepatic steatosis [34], forming a vicious cycle. Adipokine imbalance further results in IR in the liver, impaired vascular homeostasis, and low systemic inflammation [35], all contributing to AF. Serum UA concentration is closely related to systemic IR, which induces atrial structure and electrical remodeling as well as abnormal intracellular calcium homeostasis, resulting in increased susceptibility to AF [36]. An animal experimental study proved that lowering the UA level of obese mice can reduce the infiltration of macrophages and the expression of TNF-α in adipose tissue, thus reducing IR [37].

UA induces OS in mitochondria [38]. OS induces cytokine activation, lipid peroxidation, and overproduction of reactive oxygen species (ROS) [39]. ROS induces increased release of matrix metalloproteinases (MMPs) in cardiac cells [40,41]. MMPs is a crucial enzyme family involved in atrial fibrosis. Atrial fibrosis is accompanied by increased turnover and deposition of matrix proteins, resulting in uneven atrial electrical conduction and the creation of electrical reentry circuits, leading to AF. In addition, ROS also participates in the process of promoting fibrosis from fibroblasts to myofibroblasts [42,43].

The release of inflammatory cytokines and adipokines from liver adipose tissue can lead to liver inflammatory damage and systemic inflammatory response. Nod-like receptor protein 3 (NLRP3) inflammasome is activated by UA, inducing macrophages, monocytes, and endothelial cells to release a variety of inflammatory factors, such as TNF-α, interleukin-6 (IL-6), and IL-1β, causing inflammatory response [44–46]. These inflammatory factors can directly or indirectly promote cardiomyocyte apoptosis, fibrosis, and electrophysiological changes in the heart, resulting in AF. Studies have also found that the renin-angiotensin-aldosterone system (RAAS) can be activated by UA partly through the MAPK pathway, contributes to the increased release of cytokines and induces atrial inflammation [47,48].

The occurrence of AF caused by elevated UA is related to activation of ERS. When the homeostasis of the internal environment is affected by various factors that exceed the processing capacity of the endoplasmic reticulum, cells begin to experience ERS [49,50]. The cardiac endoplasmic reticulum plays a very important role in regulating myocardial excitation-contraction coupling. ERS causes cardiac fibrosis, hypertrophy, and apoptosis [51–54]. Cardiomyocyte apoptosis leads to electrical and structural remodeling of the heart, contributing to the development and maintenance of AF. UA triggers oxidation and ERS by mediating endothelial nitric oxide synthase (eNOS) activity and NO production, which induces endothelial cell apoptosis and endothelial dysfunction [55]. Besides, UA promotes cardiomyocyte apoptosis and cardiac diastolic dysfunction by activating calpain-1 and ERS, and further studies have demonstrated that the ERS inhibitor taurine deoxycholic acid (TUDCA) attenuates UA-induced apoptosis [50]. A potential target for alleviating cardiac remodeling in AF may be inhibiting ERS.

HDL-C is also involved in metabolic disorders [56]. However, patients with NAFLD show reduced levels of HDL-C [57]. HDL-C can promote cholesterol efflux, thereby exerting antioxidant and anti-inflammatory properties [58], and HDL-C also possesses antithrombotic and endothelial cell-protecting properties [59]. According to a previous study, higher levels of HDL-C were related to a lower AF risk after multifactorial adjustment [60]. Our study also found lower HDL-C levels in the AF group than non-AF group. The pre-inflammatory state due to elevated serum UA levels also affects HDL-C’s ability to reduce carotid artery damage [61], which may be related to HDL-C dysfunction caused by high UA levels [62]. In conclusion, the interaction of UA and HDL-C may contribute to CVD progression by increasing OS, inflammatory responses, and impairing endothelial cell function.

Identifying AF risk factors and predictors may help clinicians identify patients at higher risk of AF, thus contributing to early detection of AF. The clinical utility and application of our findings include that elevated UHR levels are a risk factor for the development of AF and may serve as an appropriate biomarker. UHR levels are a simple and easily obtained parameter that can be used in clinical practice to predict the risk of AF in patients with NAFLD. It is recommended that clinicians take note of evaluating this indicator when managing patients with NAFLD.

There are some limitations to the study. Firstly, the diagnosis of fatty liver in this study was based on ultrasound rather than liver puncture biopsy. Although liver puncture is the gold standard for the diagnosis of fatty liver, it is difficult to popularize and perform as an invasive test in the population, whereas ultrasound is commonly used in the clinic due to its simplicity and reproducibility. Secondly, serum UA levels are also affected by other confounding factors such as high purine diet, drug use, and genetic risk, which our study does not incorporate. Finally, the results only show that UHR is related to AF risk in NAFLD patients, but cannot prove direct causality due to the retrospective study design. Further prospective studies are needed to prove the value of UHR in predicting AF occurrence in NAFLD in multicenter prospective studies.

## Conclusion

Our study demonstrates that UHR was independently associated with the occurrence of AF in NAFLD patients. UHR as a marker of metabolic disorders and inflammation can be used to assess the risk of AF occurrence in patients with NAFLD.

## Supporting information

S1 Data (XLSX)
